# Community Volunteers and Primary Care Providers Supporting Older Adults in System Navigation: A Mixed Methods Study

**DOI:** 10.5334/ijic.5978

**Published:** 2022-03-02

**Authors:** Jessica Gaber, Stephanie Di Pelino, Julie Datta, Samina Talat, Tracy Browne, Sarah Marentette-Brown, Sivan Bomze, Pamela Forsyth, Doug Oliver, Tracey Carr, Dee Mangin

**Affiliations:** 1 McMaster University Department of Family Medicine, 100 Main Street West, Hamilton Ontario L8P 1H6 Canada; 2 Canadian Red Cross, 5700 Cancross Court, Mississauga Ontario L5R 3E9 Canada

**Keywords:** volunteers, primary care, older adults, system navigation, interprofessional health care teams, community-based health and social services

## Abstract

**Introduction:**

Primary care providers and community volunteers have important roles in supporting patient system navigation and utilization of community-based health and social services (CBHSS). This study aimed to explore the experiences and impacts of system navigation in a complex intervention supporting older adults.

**Methods:**

We used a convergent mixed methods design. Participants included primary care team members (n = 67), community volunteers (n = 38), and programme clients (n = 128) across six communities in Ontario, Canada. Data sources included focus groups, interviews, system navigation function survey for volunteers, CBHSS use survey for clients, and implementation data on CBHSS recommended by providers and volunteers and used by clients.

**Results:**

Results showed the different patterns of how CBHSS categories were recommended and ultimately used. Exercise-related CBHSS were both recommended and used, independence-related CBHSS were mostly only recommended with less uptake, and chronic health condition and diet/nutrition CBHSS were most often used by clients.

**Discussion:**

Primary care teams’ practice of system navigation was impacted by programme participation, including through learning about local CBHSS. However, volunteers felt more confident in tasks that did not include connecting to CBHSS. The programme did seem to result in many referrals, though the actual client uptake tended to be to more clinical rather than healthy lifestyle resources.

## Background

System navigation programmes were initially created in the United States in the 1990s to eliminate barriers to breast cancer care, specifically among marginalized women [1]. Since then, system navigation has expanded across settings, conditions, and the health care continuum from birth to death. Now applied to a wide variety of health needs, these programmes are set in primary care, hospitals, or community-based care around the world, with heavier clusters in the US and UK [2,3,4]. Evidence suggests that system navigation can increase patient health care utilization and screening, assist transitions between health care settings, increase patient engagement and activation in managing health, and improve patient psychological and social well-being [2,3,4,5,6]. System navigation programmes can also increase health care providers’ knowledge and skills in referring patients to community services, while enhancing their communication and trust with these services [6]. However, providers also often face challenges in providing connections to services due to a lack of awareness of them [7].

The key components of primary care are that it is comprehensive, continuous, coordinated, accessible, and patient-focused [8,9], attributes which are linked with better population health outcomes around the world [10,11]. In primary care, system navigation is commonly used to address fragmentation of the health care system and to provide more integrated, coordinated care [12,13]. System navigators within and outside health care settings can support a shift towards holistic, person-focused care that involves patient engagement with sectors outside the formal health care setting [4,14]. With its person focus and aim of coordinating care beyond the traditional health care system structures to provide more comprehensive care at the individual level, system navigation is particularly well suited to be a part of an ideal primary care model.

Health TAPESTRY is a primary care-based programme including trained community volunteers, interprofessional primary care teams, novel technology, and community engagement and connections, all linked through improved system navigation centered on older adult clients. The programme aims to help clients stay healthier for longer in the places where they live. Our definition of system navigation refers to “an individual or a team engaging in specific activities that include the following concepts: facilitating access to health-related programmes and services…; promoting and facilitating continuity of care; identifying and removing barriers to care; [and] effective and efficient use of the health care system.” [6], p.3). In Health TAPESTRY, both community volunteers and interprofessional primary care teams engage in some elements of system navigation. In Health TAPESTRY, as in other programmes, volunteer and professionals taking on system navigation tasks have overlapping responsibilities, with the key distinction being that a professional system navigator’s role includes tasks that use their clinical expertise, while volunteers take on more supportive roles [13].

Further understanding the experiences of individuals with system navigation roles could help to further refine primary care-based system navigation. There is also utility in exploring the actual uptake of recommended services by clients. Thus, this paper explores the experiences and impacts of system navigation in the context of the Health TAPESTRY approach. Specifically, we seek to understand: 1) the impact of system navigation as part of Health TAPESTRY on client connections and use of community-based health and social services (CBHSS) to support their health; 2) impacts related to system navigation on the interprofessional teams; and 3) volunteers’ perceptions about their experiences supporting older adults in connecting with CBHSS.

## Methods

### Design

This study used a convergent mixed methods design with quantitative and qualitative data collection. Data were collected in parallel, with both types collected at various time points throughout the evaluation, with equal emphasis [15]. Results were later converged to fully understand the experiences and impact of system navigation as part of Health TAPESTRY.

### Setting and Participants

The study took place in six communities across Ontario, Canada (see Appendix A for a description of these six communities). Participants included primary care team members (health care providers, clinical managers [individuals in managerial positions or executive directors], and administrative staff), Health TAPESTRY volunteers, and clients. The primary care practices involved in the study were all family health teams (FHT). FHTs are physician-led primary care practices with embedded interprofessional care providers and are a common model for providing primary care in the province [16]. This study received ethics approval from the Hamilton Integrated Research Ethics Board (#3967). All participants provided informed consent.

### The Health TAPESTRY Intervention

**Figure 1:**
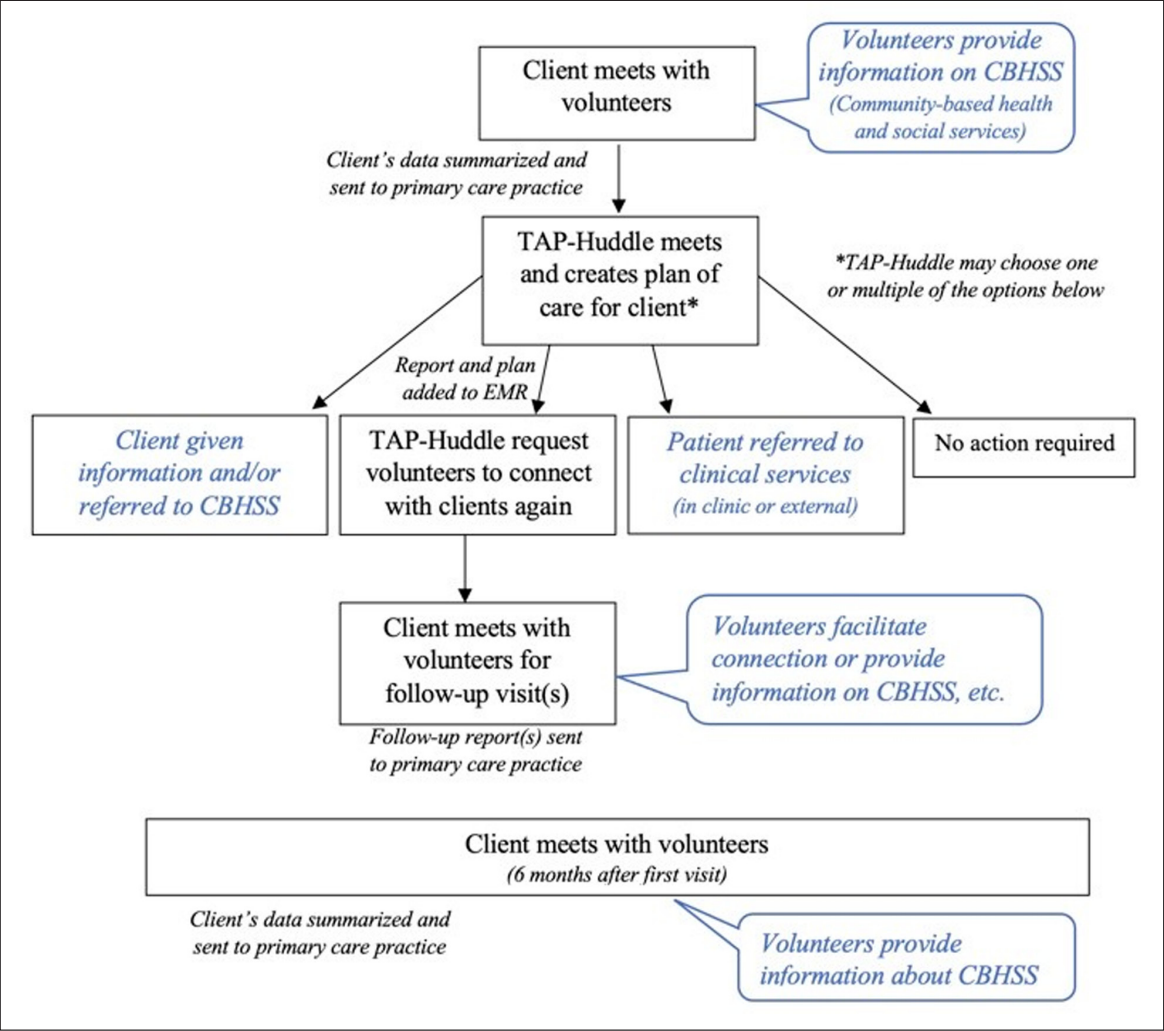
Flow of client through Health TAPESTRY.

In Health TAPESTRY, clients are visited in their homes by trained volunteers to discuss health and life goals and identify any health or health-related needs (***Figure 1***). This information is collected on tablet computers using the TAP-App, a purpose-built web application, and shared with clients’ primary care teams via an auto-generated TAP-Report. Within each primary care team there is a smaller group of interprofessional health care providers and administrative staff (approximately three-eight people per site), who meet weekly to discuss clients in the programme. This smaller group is referred to as the ‘huddle’. The huddle operates slightly differently at each site, however in general, the huddle will review the TAP-Report for each new Health TAPESTRY client and discuss potential areas of concern and steps to address those concerns. Clients’ charts are often reviewed in conjunction to gain additional information. Based on the discussion, the huddle creates individualized plans of care for clients. The clients’ family physicians may or may not be part of the huddle (site and schedule dependent), however the plan of care is documented in the electronic medical record. These plans of care may include referrals to CBHSS. These referrals may be organized directly by any members of the primary care team (i.e., making the referrals themselves), or they can fill out a form on the TAP-App (with eight potential follow-up actions and an ‘Other’ option, see Appendix B) to send volunteers back to clients on a follow-up visit. After these follow-up visits, a Follow-Up Report (Appendix C) is generated and sent to the primary care team. Regardless of follow-up action, clients are visited again by volunteers six months after their initial visit, and a 6-Month TAP-Report is created.

### Data Collection and Measures

Data were collected at various time points as implementation was not simultaneous across the six communities. Health care providers and clinic managers were invited by email and participated in focus groups (for providers) or interviews (for managers and individual providers as needed for scheduling) after ten 6-month TAP-Reports had been seen by that clinical site (December 2018–November 2019). Volunteers were also invited by email and participated in volunteer-only focus groups or individual interviews as needed, one year after volunteers first saw clients in that site (April 2019–November 2019). Volunteers also responded to a survey when they had personally completed 12 months of participation with the programme. They were invited to participate via email through LimeSurvey (March 2019–September 2020) [17]. Client survey data were collected after six months of their enrollment in the programme (October 2018–March 2020), with selected client interviews taking place after their programme completion (February 2019–November 2019).

Qualitative data collection was completed by research staff at the Department of Family Medicine (by RC, JD, SD, JG, CK, and FP; all identified as female). Some of the health care providers were known to research staff through work on Health TAPESTRY or other programmes. Quantitative data was collected via the TAP-App for clients and through both LimeSurvey and the TAP-App for volunteers.

Focus groups and interviews for health care team members and volunteers were conducted using a semi-structured interview guide developed by the research team and adapted for each respondent group. The question guide was created to address multiple research objectives, some of which are not covered in this study and are reported elsewhere. The overall focus of the questions was programme improvement and included understanding the implementation of Health TAPESTRY, integration of Health TAPESTRY into practice, impacts and outcomes, and resources. Focus groups were one-hour long, conducted by two or more members of the research team, and held at the clinical sites (for health care providers) or a community location (for volunteers). Interviews with these groups, which were conducted by a solo research team member, were either done in person or over the phone depending on interviewee availability and ranged from 18–74 minutes.

Client phone interviews ranged from 30–45 minutes with a single research team member. Client interviews also used a semi-structured approach with question categories including their understanding of the programme, how their care was impacted, and their engagement with community resources. No participants were compensated but light refreshments were provided for focus groups. All focus groups and interviews were digitally recorded and transcribed by an external transcriptionist.

Volunteers completed a system navigation function survey, an 18-item scale that measured their confidence in fulfilling the related functions of their role. The items in the survey were based on the Health TAPESTRY volunteer job description and the checklist by Duggleby et al. [18]. Responses were on a 6-point scale and ranged from 0 (“not at all confident”) to 5 (“very confident”). Volunteer demographics (age, gender, education, ethnicity, and years of volunteer experience) were also collected.

Clients completed the Community and Social Service Use Survey [19,20] which was administered by volunteers on the TAP-App during clients’ final (6-month) home visit. The survey gained an understanding of clients’ engagement with community resources during their involvement in the programme by asking about their use of twelve different categories of community resources.

Lastly, data pertaining to community resource follow-up requests by the clinical team, and volunteer follow-up actions conducted, including detail from the Follow-up Reports, were exported from the TAP-App by research staff.

### Data Analysis

Qualitative analysis of focus group and interview data was completed at a semantic level using Braun and Clarke’s thematic analysis steps [21]. JG and FP developed an initial high-level coding structure based on the focus group guide questions, then familiarized themselves with the data. The first few transcripts were coded more deductively to understand how this coding structure fit the dataset with remaining transcripts coded at a more inductive level, open coding below the high-level categories in the initial coding structure. This coding was conducted by one of three team members (SD, JG, and FP) and then checked by another coder to ensure consistency. All coders were familiar with Health TAPESTRY as they were involved in both implementation and evaluation of the programme. This analysis team, with occasional inclusion of RV, held regular meetings to review the coding structure, search for themes and review them, re-organize codes into the categories and themes as reflected by the data, and reach consensus on these themes and sub-themes. The analysis was done using NVivo 12 [22].

Analysis of qualitative volunteer follow-up action data was completed through inductive coding by SD and JG and subsequently grouped into categories of follow-up action based on a deep read of the data.

For the system navigation survey completed by volunteers [18], means and standard deviations were calculated for each question. For the client survey [19,20], responses were recategorized (by SD and JG) into the twelve community resource categories; frequency counts per category were determined. Frequency counts were also gathered for follow-up actions requested by the interprofessional team. Demographics were calculated for all participant groups. Analyses were completed in SPSS 26 [23] or Microsoft Excel 2009.

## Results

### Participants

Overall, there were 233 individual participants (67 health team members, 38 volunteers, and 128 clients) with data included in this manuscript. There were 67 health team members who participated in a focus group or interview, see ***Table 1*** for their professions. There were 38 volunteers who completed the volunteer system navigation function survey; they ranged in age 18 to 78 years. Most of the volunteer participants, as in the volunteer pool at large, were female and European/white.

**Table 1:** Details about Participants Involved.


PROFESSIONS OF HEALTH CARE TEAM MEMBERS IN FOCUS GROUPS AND INTERVIEWS	N (%)	

Physician	23 (34.3)	

Manager or Director	9 (13.4)	

Registered Nurse	7 (10.4)	

Occupational Therapist	5 (7.5)	

Nurse Practitioner	5 (7.5)	

Dietitian	4 (6.0)	

Social Worker	4 (6.0)	

Pharmacist	3 (4.5)	

System Navigator	2 (3.0)	

Administrative Assistant	2 (3.0)	

Physician Assistant	1 (1.5)	

Registered Practical Nurse	1 (1.5)	

Physiotherapist	1 (1.5)	

**VOLUNTEERS IN THE SYSTEM NAVIGATION SURVEY**	**N = 38**	

Age (years)		

Range	18–78	

Mean (SD)	46.74 (21.1)	

Gender*		

Female, n (%)	28 (80.0)	

Male, n (%)	7 (20.0)	

Ethnicity*		

European/white, n (%)	5 (55.6)	

South Asian, n (%)	2 (22.2)	

African/Black, n (%)	1 (11.1)	

Other, n (%)	1 (11.1)	

Highest level of education*		

Bachelor’s, n (%)	11 (30.6)	

Master’s, n (%)	7 (19.4)	

Enrolled in Bachelor’s, n (%)	7 (19.4)	

Community college, n (%)	6 (16.7)	

High school, n (%)	3 (8.3)	

Professional degree, n (%)	2 (5.6)	

Years of volunteer experience		

Range	0–40	

Mean, SD	11.2 (11.0)	

	**CLIENTS WHO COMPLETED THE COMMUNITY SERVICE USE SURVEY N = 110**	**CLIENTS IN INTERVIEWS N = 39**

Gender* (N = 108)		

Female, n (%)	75 (69.4)	25 (64.1)

Male, n (%)	33 (30.6)	14 (35.9)

Ethnicity* (N = 108)		

European/White, n (%)	102 (94.4)	35 (89.7)

Other, n (%)	3 (2.8)	3 (7.7)

African/Black, n (%)	1 (0.9)	1 (2.6)

Indigenous, n (%)	1 (0.9)	0 (0.0)

Don’t know, n (%)	1 (0.9)	0 (0.0)

Marital Status (N = 110)		

Married, n (%)	55 (50.0)	18 (46.2)

Common law, n (%)	3 (2.7)	0 (0.0)

Divorced, n (%)	15 (13.6)	3 (7.1)

Widower, n (%)	29 (26.4)	13 (33.3)

Single/never married, n (%)	6 (5.5)	5 (12.8)

No answer, n (%)	2 (2.0)	0 (0.0)

Household Income (N = 110)		

$20,001-$50,000, n (%)	43 (39.1)	17 (43.6)

$50,001-$70,000, n (%)	24 (21.8)	6 (15.4)

$70,001-$100,000, n (%)	18 (16.4)	8 (20.5)

Under $20,000, n (%)	8 (7.3)	1 (2.6)

$100,001-$150,000, n (%)	7 (6.4)	5 (12.8)

Greater than $150,000, n (%)	2 (1.8)	0 (0.0)

No answer, n (%)	8 (7.3)	2 (5.1)


Percentages are based on valid responses. * Though other options were provided, only those options that had data are listed in this table; some had multiple missing options.

Of the 252 clients who completed the Community and Social Service Use Survey, 110 stated they had received information about one or more community resources; these are the clients we included in this paper. Their ages ranged from 70 to 97 years, with the mean age being 76.8 years (SD: 5.4). The majority (94.4%) identified as European/white, half were married (50.9%), and 69.4% identified as female. Of all enrolled clients, 39 were invited to a one-on-one interview (21 overlapped with the survey clients). The demographics of these individuals, and all other participants involved are in ***Table 1***.

### Understanding client connections to and use of CBHSS

#### Follow-up requested of volunteers by interprofessional teams

The most requested follow-up actions that interprofessional teams requested of volunteers were for volunteers to connect a client to a specific community programme (42.6%) or learn about clients’ interests (24%). ‘Other’ actions made up 16.3% of requests. The most common ‘Other’ actions were providing information about an exercise programme, services for living safer and more independently at home, educational resources, and financial resources. See ***Table 2*** for further follow-up requests.

**Table 2:** Interprofessional Team Member Follow-Up Action Requests to Volunteers.


FOLLOW-UP ACTIONS REQUESTED	N (%)

Facilitate connection to a specific community programme	55 (42.6)

Learn more about their interests and connect to community programmes and services	31 (24.0)

Other	21 (16.3)

Check-in on their progress toward life/health goals	12 (9.3)

Accompany the client to a community programme or service	3 (2.3)

Complete an additional clinical screening tool	2 (1.6)

Refer to Canadian [Name] transportation services	2 (1.6)

Review care plan instructions with client	2 (1.6)

Follow-up on a referral with client in [time period] (e.g., follow up in 3 months on suggestion for client to visit an exercise programme)	1 (0.8)


#### Follow-up carried out by volunteers

After volunteers received follow-up requests from the interprofessional team, they conducted follow-up visits. As reported in the Follow-up Reports, the most common action completed by volunteers was to provide clients with resources (e.g., written materials such as pamphlets explaining the programmes or services, or contact information), which aligns with the most common request from the huddle. The resources they provided primarily pertained to exercise and recreation programmes, such as a booklet containing recreation centre fitness programmes, or a list of exercise/fitness programmes run by the primary care clinic. Resources to improve client’s independence at home, such as home help and fall prevention classes, along with setting clients up with digital personal health records were also common. Less common but still notable requests included providing information about transportation options and social activities within the community. When volunteers provided clients were resources, it could have been for one or multiple programmes and services depending on the request; most commonly, they conducted the follow-up visit focused on a single issue and referral, yet sometimes also discussed other programmes or left other printouts. Further, volunteers would often provide explanations of the programmes and services to the clients and learn what options the clients were most interested in. Rather than providing clients with resources, some follow up visits were intended for volunteers to check-in regarding the resources that were mailed to clients, or to assist in filling out forms (e.g., self-referral forms) or making phone calls with clients. The decision to provide resources versus a check-in was made at the discretion of the client’s primary care team.

Volunteers did note for some clients that they appeared to already be supported by family and friends, were managing their health well, and were working on the goals that volunteers had helped them establish during the initial visit. However, common gaps or needs that volunteers noted were that clients were often struggling with transportation, home management, or independence. Volunteers perceived that some clients may have been experiencing loneliness or lack of socialization, and some had concerns about their mental or emotional health.

#### Client understanding of resources provided

The most common categories of community programmes or services that Health TAPESTRY clients said they were *given information* about were ‘Chronic Health Conditions’ (15.2%), ‘Diet and Nutrition’ (13.1%), and ‘Fit and Active’ (11.4%). ‘Caregiving’ and ‘Volunteering’ were least common. ***Table 3*** shows the data for the remaining nine categories.

**Table 3:** CBHSS Categories that Clients were Given Information About.


CATEGORY	N (%)

Chronic health conditions, e.g., education or assistance for those living with chronic health conditions, referrals to providers for specific conditions	44 (15.2)

Diet and nutrition, e.g., referrals to dietitians, cooking classes, meal delivery services	38 (13.1)

Independence at home, e.g., fall prevention course, snow removal	37 (12.8)

Fit and active, e.g., exercise and walking programmes	33 (11.4)

Seniors centres and programmes for healthy aging without a specific programme suggestion	33 (11.4)

Counselling and friendly visiting	21 (7.2)

Educational, e.g., local university programmes	14 (4.8)

Online health information, i.e., links to trustworthy online places to find health information	9 (3.1)

Social, e.g., social clubs, coffee clubs	9 (3.1)

Transportation programmes	9 (3.1)

Creative, e.g., classes for art, knitting, music	5 (1.7)

Caregiving supports	4 (1.8)

Volunteering opportunities	2 (0.7)

Not stated	32 (11.0)


#### Actual client uptake of CBHSS

The most common categories of community programmes or services that Health TAPESTRY clients said they *attended or used* were ‘Chronic Health Conditions’ (27.3%), ‘Diet and Nutrition’ (17.4%), and ‘Fit and Active’ (10.7%). ‘Caregiving’ (0.8%) and ‘Transportation’ (0.8%) were among the least common categories which clients stated they attended or used. ***Table 4*** shows the data for the remaining categories.

**Table 4:** CBHSS Categories that Clients Said They Attended or Used.


CATEGORY	N (%)

Chronic health conditions, e.g., education or assistance for those living with chronic health conditions, referrals to providers for specific conditions	33 (27.3)

Diet and nutrition, e.g., referrals to dietitians, cooking classes, meal delivery services	21 (17.4)

Fit and active, e.g., exercise and walking programmes	13 (10.7)

Counselling and friendly-visiting	12 (9.9)

Seniors centres and programmes for healthy aging without a specific programme suggestion	12 (9.9)

Independence at home, e.g., fall prevention course, snow removal	11 (9.1)

Online health information, i.e., links to trustworthy online places to find health information	7 (5.8)

Creative, e.g., classes for art, knitting, music	3 (2.5)

Educational, e.g., local university programmes	3 (2.5)

Social, e.g., social clubs, coffee clubs	2 (1.7)

Caregiving supports	1 (0.8)

Transportation programmes	1 (0.8)

Volunteering opportunities	0 (0.0)


#### Converging data about client connections to and subsequent uptake of CBHSS

Exercise was the most frequent category of primary care provider suggestion. It was also the most common category volunteers reported they gave clients information about (see ***Table 5*** for a summary of how these data points converged). In contrast the most common category of services clients reported they got information about and most commonly used, was chronic health conditions, followed by diet and nutrition.

**Table 5:** Top Three Categories of CBHSSs Mentioned at Each Step.


	INDEPENDENCE	FIT & ACTIVE	CHRONIC HEALTH CONDITIONS	DIET & NUTRITION	EDUCATION & FINANCES	PERSONAL HEALTH RECORD

Interprofessional Team	2^nd^	1^st^			3^rd^	

Volunteers	2^nd^	1^st^				3^rd^

Clients – Information Given	1^st^		3^rd^	2^nd^		

Clients – Attended		3^rd^	1^st^	2^nd^		


Independence at home followed as the second most common category for both provider recommendations and volunteer reports of resources provided. However, independence was the third most common category clients reported they received information about, with substantially fewer clients indicating they used this information. Though the interprofessional team suggested resources for finances and educational resources, this did not show up again through volunteers and clients. Despite how frequently volunteers noted setting clients up with digital personal health records, this was not among the most common resources that clients reported being offered or using, nor was it requested by providers in the Follow-Up Reports.

#### Client experiences of being connected to CBHSS

Regardless of attendance, clients stated that they learned about programmes they were not previously aware of, with some clients sharing these resources with others in the community, although some felt the information was redundant, as they were already attending or aware of the services that were offered to them.

Despite some clients not being interested in the information, many expressed that it was good to know that opportunities were available if or when they may need them in the future.

“I just sum it up in one thing, that we got a lot of peace of mind about it” (Client 1).

Some clients felt as if the programme impacted their ability to navigate the system.

“I’m much happier with the system, whether it’s due to the family doctor or not, that was probably part of it, but it just seems much easier to be able to navigate the [health] system now” (Client 2).

Some clients said their ability to navigate the system did not change. Generally, this was because they felt they did not need to navigate the system during this time or were already navigating the system well.

### Impacts on the interprofessional teams resulting from system navigation

The interprofessional teams in the primary care clinics reported that they learned about community programmes and resources, and their ability to connect patients and their practice of system navigation were impacted in a positive way.

“I wasn’t aware of those services that were out there to mention to patients and now I have that knowledge and can pass it on—and how to navigate it.” (Huddle member 1)

In learning about these community programmes and resources, the interprofessional teams realized what barriers clients may face in accessing these services, which enhanced their troubleshooting in finding solutions to these barriers.

“It has again opened the eyes of many providers as to what … barriers are out there, what kind of supports people need apart from their own service, and has just provided a better holistic view of how to better support patients.” (Clinic manager 1)

Interprofessional team members described that the huddle approach made system navigation feel less daunting.

“It also feels less daunting; [usual care]… is a lot more daunting than to be able to sit around the team and know that each person at the table also heard what you’ve heard and they’re going to follow up in their own timeline.” (Huddle member 2)

The programme helped to define the teams’ practice of system navigation.

“I think it has broadened our navigation… Health TAPESTRY has allowed us to expand on that collaboration and with other organizations that we typically don’t partner with; truly bring that kind of health and social together to provide good outcomes.” (Clinic manager 2)

The huddle team making connections to CBHSS helped the family physicians, and the new knowledge of CBHSS that primary care providers in Health TAPESTRY gained was then available to be shared with other providers in their teams.

“It was a little bit difficult getting started because we weren’t as aware of the different services that we could connect clients with. But now that we have become more familiar with the community supports that are out there, I have providers reaching out to me for clients that aren’t involved with Health TAPESTRY to say, like ‘Hey, I don’t really know where to send this person, this is what they’re looking for.’” (Huddle member 3).

Health care providers engaged more with community programmes and services. There was also a perception among providers that Health TAPESTRY helped facilitate access to CBHSS for enrolled clients.

“It’s… the difference between somebody having to come in and request something versus somebody calling you and saying ‘Do you want this?’” (Physician 1)

Health care providers identified gaps and local issues in their communities, such as transportation and a lack of programmeming, and began to address these issues.

“Finding services, especially those that are free, as our senior population is usually on a fixed income… That has been a challenge at times. There seems to be a service gap in programmeming for seniors, so working with this programme actually led me to connect with [Public library], who did have some funding to run some senior programmeming.” (Huddle member 2)

### Volunteers’ experiences of system navigation: supporting older adults in connecting with CBHSS

Every item on the questionnaire [18] where volunteers indicated their confidence on the system navigation-related aspects of their role had a relatively high average, with the lowest average score being 4.5 out of 6. The aspects where they had the most confidence were: knowing when to access their volunteer coordinator, reporting to the primary care team about client needs or concerns, managing critical or urgent issues on home visits, and identifying client barriers to accessing CBHSS. Aspects of their role where volunteers had slightly less confidence or were more variable in their confidence included: coordinating access for clients to transportation services or other specific programmes that were found through online tools or were identified by the primary care team. Volunteers were also not quite as confident in their roles around educating clients about the use of trusted resources for health information (***Table 6***).

**Table 6:** Volunteer Confidence in Undertaking System Navigation-Related Aspects of their Role (N = 38).


SYSTEM NAVIGATION FUNCTIONS	MEAN (SD)

Know when to access my volunteer coordinator	5.8 (0.4)

Report to the primary care team on any concerns from home visits or potential needs of clients	5.6 (0.7)

Manage any critical or urgent issues that arise while on a home visit	5.2 (0.8)

Identify client barriers to accessing community programmes and services	5.2 (0.8)

Facilitate setting SMART health and life goals with the client	5.1 (0.9)

Identify client needs for community-based health and social services and programmes	5.1 (0.9)

Receive and review any follow up instructions from the primary care team with the client	5.1 (1.1)

Report to the primary care team on client barriers, enablers, and potential areas of interested related to community programmes and services	5.0 (0.9)

Assist clients in overcoming barriers to accessing community programmes and services	4.8 (0.9)

Follow-up on client progress toward health or life goals	4.8 (1.2)

Assist clients in finding community programmes and services that meet their interests and needs (e.g., through Canada211, local community service directives, or thehealthline.ca)	4.8 (1.1)

Assist clients in reaching out to community services for further information or to address access issues	4.7 (1.3)

Use motivational interviewing techniques to support client in goal attainment	4.7 (1.1)

Educate about and encourage use of trusted resources for health information, such as the [Name] Optimal Aging Portal	4.6 (1.2)

Follow-up on uptake of services identified to be of interest to be of interest to the client or recommended by the primary care team	4.6 (1.3)

Create linkages between the client and community programmes and services such as [Name] programmes, programmes found through online tools, or those identified by the primary care team	4.6 (1.2)

Coordinate client access to needed services, such as through the [Name] Transportation programme or local transit	4.5 (1.4)


Responses for each item were on a 6-point scale: 0 (not at all confident) to 5 (very confident).

## Discussion

The aim of this paper was to explore the experiences and impacts of system navigation in the context of the primary care-based Health TAPESTRY approach. The follow-up actions that interprofessional team members could request volunteers carry out with clients can all be linked to the key components of primary care. Connecting clients to community programmes and completing additional screening tools augments the comprehensiveness of care; following-up on client goals extends continuity of care and is person-focused, as is learning more about client interests; access and coordination could be improved through accompanying clients to CBHSS referring them to transportation services [8,9]. In specifically seeking to understand the impact of system navigation on client connections and use of CBHSS to support their health, the patterns across CBHSS that each group (providers, volunteers, and clients) said they’d given, been given, or attended was not consistent. In such a pragmatic, community-based programme, this is not particularly surprising. Referring individuals to the most appropriate programmes can be difficult and following through to actually attend can be even harder.

One of the inconsistencies that arose was between two categories of CBHSS, diet/nutrition and chronic health conditions. They were among the most used only as reported by clients. The high use of diet and nutrition programmes and services (which included dietitian referrals) may have been due to three factors: 1) the sensitivity of the nutrition scale (SCREEN-II) used; 2) the technical issues experienced with this survey, which reported a number of clients to be at high nutritional risk when in fact, they were not; 3) the regular attendance of dietitians in huddle meetings. Notably, the needs associated with both diet/nutrition and chronic health conditions may be better served with clinical expertise as they need health promotion and self-management services, both of which are roles more commonly taken by professional system navigators rather than volunteers [13]. Therefore, these services may not be reflected in the follow-up actions asked of or completed by volunteers, but managed internally within the primary care clinic and clients saying they attended more diet/nutrition programmes may have reflected their use of internal primary care resources. This may also explain, in part, the differences in that providers and volunteers said they most often requested or recommended fitness programmes, while clients said they most often received chronic health programmes. The data in this study do not include what providers said directly to clients – just what they asked of volunteers to follow up with – so they may have been requesting that volunteers follow up with exercise, while they themselves followed up to discuss chronic conditions.

Health TAPESTRY did take place in six different communities with different community sizes, family health team sizes, and number and breadth of resources (as per Appendix A). While this paper did not focus on distinctions between communities, there were some distinctions. Two of the six communities made up nearly 70% of the clients who said they had received information about programmes. While one of these communities was the largest, urban city in the programme, the other was mid-range in population size, in a mostly rural county; however, volunteers were particularly encouraged to support clients in connecting to CBHSS at this site. At least one client in each of the six communities were referred to programmes about diet/nutrition, while fitness and activity, chronic health conditions, and counselling/friendly visiting had at least one client from five of the communities. Referrals to programmes for caregiving support and transportation were only seen in the larger communities.

Overall, participating in Health TAPESTRY appeared to clarify the process of system navigation and specifically the use of CBHSS at the interprofessional primary care team level. Providers’ awareness of local programmes and services increased, which is often a challenge providers face in offering these connections [7]. This increase in awareness also resulted in learning spread throughout the team. The interprofessional team learned from one another, with family physicians learning as their patients were referred, and non-Health TAPESTRY health care providers learning as other providers shared their knowledge of CBHSS. When faced with boundaries in patients accessing CBHSS, providers attempted to aid in reducing barriers to services or treatments, which is a fundamental role of professional system navigators [13,24].

Volunteer roles related to system navigation usually consist of performing such tasks as liaising between providers and finding resources for unmet needs, rather than areas where a provider’s clinical knowledge would be needed [13]. Health TAPESTRY volunteers’ expressed confidence in connecting with primary care teams about client needs, but were somewhat less confident in the follow-up, coordination, and linking of clients to CBHSS, though even the lowest average confidence level in any volunteer role item was still 4.5 out of 6. Since volunteers did still manage to link clients to CBHSS, this indicates that there may be a potential for further coaching in this area to close the confidence gap. However, due to the clinical nature of many of the programmes or services (either community-based or within the clinic environment itself), the programmes and services that clients were most likely to engage with in Health TAPESTRY were often beyond the scope and role of Health TAPESTRY volunteers. The volunteers still played an important role in connecting clients to programmes and services however, as they were the link between the client and the primary care team, a link further facilitated by the technology used in the programme.

In Valaitis et al. [6], the authors described three actions that influenced the start-up and maintenance of system navigator programmes: “a) improve delivery of health and social care services; b) support and manage specific health needs or specific population needs, and; c) improve quality of life and wellbeing of patients” (p. 5). Using these to define success in system navigation in this programme: a) more connections were made between the formal health care setting and CBHSS; b) specific health wants and needs were identified through Health TAPESTRY and managed both through referrals to CBHSS and in-house referrals through the primary care team. Potential effectiveness in impacting clients’ quality of life and wellbeing will be addressed in other papers.

### Strengths and Limitations

A major strength of the research design was the inclusion of multiple different stakeholder groups at multiple timepoints including providers from a variety of disciplines, volunteers, and clients, fostering rigour through source triangulation as well as more potential generalizability to other programmes with multiple stakeholders. These perspectives were captured using different types of data collection methods and administered by several different groups spanning six distinct communities in Ontario, further increasing our triangulation of methods and settings.

We acknowledge limitations of this study. First, the data collected represents only a subset of the entire pool of providers, volunteers, and clients in the programme. Of the providers and volunteers who participated in interviews or focus groups, only a subset self-selected to participate in focus groups or surveys. The majority of volunteers and clients who provided data were female, 80% and 75% respectively, which may impact generalizability, though there were more female volunteers and clients in the programme. Many volunteer programmes tend to have more female volunteers [25]. Further, a majority of the clients in this study identified as European/white also limiting the generalizability of this study; this reflected the ethnic makeup of the overall programme. Ethnicity and gender-based data was not collected for providers, though most were physicians which could have limited the diversity of our interprofessional pool. While the diversity of sites was a strength of the research, it could also be considered a limitation in how the programme ran, as each FHT had a different group of professionals who were directly engaged in making client care plans, which may have resulted in different CBHSS being suggested and eventually used by clients. Lastly, clients reported their community service use to volunteers, which may have biased client responses.

## Conclusions

Making connections to community-based health and social services can be difficult, and actual client uptake of these CBHSS can be even more difficult to help facilitate. Results of this study showed the pattern of how different groups involved in the Health TAPESTRY intervention (primary care providers, community volunteers, and older adult clients) suggest, recommend, or actually use programmes and services. Each point in the process has interesting distinctions. Many of these seemed to revolve around the distinction between roles that a health care professional versus a volunteer can take in system navigation. This was further reflected in the roles the volunteers felt the most comfortable in – with less comfort surrounding roles that required them to connect clients to CBHSS – and the impacts on the health care teams, who said they learned more about local CBHSS through programme implementation. A substantial amount of the programmes and services that clients said they actually attended were more clinical, rather than the healthy lifestyle and wellbeing resources that a community volunteer could just as easily refer clients to. This may be that the programmes clients actually attended were based on their more defined, clinical, health needs rather than related to general wellbeing goals, or perhaps that the clinical teams were driving client uptake of programmes; clients also may have been more likely to take suggestions from a clinical team they knew and had a longer-term trusting relationship with. Regardless, this programme did seem to result in many referrals to programmes and services, whether they were community-based or inside the clinical setting.

## Additional Files

The additional files for this article can be found as follows:

10.5334/ijic.5978.s1 Appendix A. Descriptions of the six communities that Health TAPESTRY was implemented in.

10.5334/ijic.5978.s2 Appendix B. Health TAPESTRY Volunteer Functions Related to System Navigation.

10.5334/ijic.5978.s3 Appendix C. A sample Follow-up Report for a fictional client.
