# Supplementary material for: Community Volunteers and Primary Care Providers Supporting Older Adults in System Navigation: A Mixed Methods Study
Source: Int J Integr Care. 2022 Mar 2;22(1):18. doi: 10.5334/ijic.5978 (PMC8896251; doi:10.5334/ijic.5978)
Supplement: Appendix A. — Descriptions of the six communities that Health TAPESTRY was implemented in. [file ijic-22-1-5978-s1.pdf]

Appendix A: Descriptions of the six communities that Health TAPESTRY was implemented in.

Dufferin county is a predominantly rural community consisting of three towns with a population of 61,735.[1] Approximately 15% of the population is over 65 years of age, which is comparable to the provincial average of 16.7%).[1] Recently, Dufferin became an age-friendly community meaning they have implemented different services, structures and policies to become more inclusive of older adults.[2] Seniors in the community can participate in recreation and other programming through local community and senior centres. Practical support programs are also available in the community, but are limited and often come at a cost. Dufferin residents have access to multiple walk-in clinics, an urgent care centre and a hospital for medical care. Residents can travel to larger surrounding regions to access services as well. Currently, there is no community wide transportation system, however the largest town does have a small bus system.

Hamilton is a large urban community, the third largest city in Ontario, Canada with a population of 535,917.[3] The senior population is just over the provincial average at 17.3%.[3] Hamilton is considered an age-friendly community. The city has a wide variety of programs and services available to seniors that are free or low cost. The services include recreation, financial support, assistance with meals, groceries or transportation, among others. Residents have access to multiple hospitals, urgent care centres and walk-in clinics to receive medical assistance. Hamilton has an extensive bus public transportation system, and additional services to assist individuals unable to use regular bus transportation.

Harrow is a very rural community (population 2,710) with an above average senior population of 19.9%.[4] Harrow is surrounded by a number small communities which residents travel to for community programs and services that Harrow may not have. These programs and services are offered at community centres, libraries and senior centres. The town of Essex is not classified as an age-friendly community. Within the community, there is no hospital, walk-in clinics or urgent care centres. Residents would need to travel outside of Harrow to access medical services other than at Harrow Health Centre (a family health team). Harrow does not have a public transportation system, and the distance to other towns with services the residents may need to access can be far.

Niagara-on-the-Lake (NOTL) is a rural community with a population of 17, 511.[5] NOTL has a large number of seniors, with 30% of the population over the age of 65 years.[5] Recently, NOTL became an age-friendly community. Seniors can participate in recreation and other service programs through municipal and regional organizations. There are some supports available for transportation, housekeeping, meal delivery in the area but there are costs associated with these services. Within NOTL, there is a walk-in clinic however to access a hospital or urgent care centre, residents must travel outside the community. NOTL does lack affordable housing in the community and is a predominantly auto-centre community with limited public transportation.

Sault Ste. Marie is a predominantly rural community in Northern Ontario with a population of 78 159.[6] Approximately 22% of the population is over 65 years of age.[6] It is bordered by two First Nations communities. Seniors in the community have access to a variety of community-base

programs for recreation, friendly visiting, meal and housekeeping services, transportation and more. Residents have access to a hospital and walk-in clinics within the site, however there is no urgent care centre. Sault Ste. Marie has taken steps to be more inclusive of older adults and is considered an age-friendly community. There is a bus-based transportation system that services the town.

Windsor is an urban city with a population of 329,144.[7] The senior population is approximately 17.2% which aligns with the provincial average of 16.7%.[7] There are a wide variety of community programs, recreation and service, offered through the city, and other community organizations. Many of the programs and services are available to seniors for free or for a low cost. Windsor has several hospitals, walk-in clinics and urgent care clinics within the city for residents to access. Windsor is considered an age-friendly community. The city has bus transportation system that services the city.

References

1. Statistics Canada. Dufferin, CTY [Census division], Ontario and Ontario [Province] (table). Census Profile. 2016 Census. Statistics Canada Catalogue no. 98-316-X2016001. 2017. https://www12.statcan.gc.ca/census-recensement/2016/dp-pd/prof/details/page.cfm?Lang=E&Geo1=CD&Code1=3522&Geo2=PR&Code2=35&SearchText=dufferin&SearchType=Begins&SearchPR=01&B1=All&TABID=1&type=0 (accessed 15-03-2021).
2. World Health Organization. Age-Friendly in Practice. https://extranet.who.int/agefriendlyworld/age-friendly-practices/ (accessed 15-03-2021).
3. Statistics Canada. Hamilton [Census subdivision], Ontario and Ontario [Province] (table). Census Profile. 2016 Census. Statistics Canada Catalogue no. 98-316-X2016001. 2017. https://www12.statcan.gc.ca/census-recensement/2016/dp-pd/prof/details/page.cfm?Lang=E&Geo1=CSD&Code1=3525005&Geo2=CD&Code2=3525&SearchText=hamilton&SearchType=Begins&SearchPR=01&B1=All&TABID=1&type=0 (accessed 15-03-2021).
4. Statistics Canada. Harrow [Population centre], Ontario and Ontario [Province] (table). Census Profile. 2016 Census. Statistics Canada Catalogue no. 98-316-X2016001. 2017. https://www12.statcan.gc.ca/census-recensement/2016/dp-pd/prof/details/page.cfm?Lang=E&Geo1=POPC&Code1=0360&Geo2=PR&Code2=35&SearchText=harrow&SearchType=Begins&SearchPR=01&B1=All&TABID=1&type=0 (accessed 15-03-2021).
5. Statistics Canada. Niagara-on-the-Lake, T [Census subdivision], Ontario and Niagara, RM [Census division], Ontario (table). Census Profile. 2016 Census. Statistics Canada Catalogue no. 98-316-X2016001. 2017. https://www12.statcan.gc.ca/census-recensement/2016/dp-pd/prof/details/page.cfm?Lang=E&Geo1=CSD&Code1=3526047&Geo2=CD&Code2=3526&SearchText=niagara%20on%20the%20lake&SearchType=Begins&SearchPR=01&B1=All&TABID=1&type=0 (accessed 15-03-2021).
6. Statistics Canada. Sault Ste. Marie [Census agglomeration], Ontario and Ontario [Province] (table). Census Profile. 2016 Census. Statistics Canada Catalogue no. 98-316-X2016001. 2017. https://www12.statcan.gc.ca/census-recensement/2016/dp-pd/prof/details/page.cfm?Lang=E&Geo1=CMACA&Code1=590&Geo2=PR&Code2=35&Searc

hText=sault%20ste%20marie&SearchType=Begins&SearchPR=01&B1=All&TABID=1&type=0 (accessed 15-03-2021).
7. Statistics Canada. Windsor [Census metropolitan area], Ontario and Ontario [Province] (table). Census Profile. 2016 Census. Statistics Canada Catalogue no. 98-316-X2016001. 2017. https://www12.statcan.gc.ca/census-recensement/2016/dp-pd/prof/details/page.cfm?Lang=E&Geo1=CMACA&Code1=559&Geo2=PR&Code2=35&SearchText=windsor&SearchType=Begins&SearchPR=01&B1=All&TABID=1&type=0 (accessed 15-03-2021).
