# Supplementary material for: Community Volunteers and Primary Care Providers Supporting Older Adults in System Navigation: A Mixed Methods Study
Source: Int J Integr Care. 2022 Mar 2;22(1):18. doi: 10.5334/ijic.5978 (PMC8896251; doi:10.5334/ijic.5978)
Supplement: Appendix B. — Health TAPESTRY Volunteer Functions Related to System Navigation. [file ijic-22-1-5978-s2.pdf]

Appendix B: [Program Name] Volunteer Functions Related to System Navigation

During initial home visits

[Program Name] volunteers build a relationship with the client and complete a series of surveys in the [Program Name]-App to learn more about what is important to them, as well as their health and life goals. In addition to this, volunteers may complete the following functions if a client is interested:

- Setting up an online personal health record (PHR)
- Assisting client in using the [Name] Optimal Aging Portal

During follow-up visits as initiated by the huddle team

Once the huddle team has reviewed the [Program Name]-Report and discussed a care plan for the client, the huddle may ask volunteers to complete follow-up actions to further support the client. These may include:

- Supporting clients to explore their interests and assisting them to connect to community programs and services that address them (e.g., using Canada211 or thehealthline.ca);
- Facilitating a connection to a specific community program as directed by the huddle;
- Reviewing care plan instructions with the client to ensure their understanding of the plan and follow-up;
- Following-up on a referral that was made for the client by the huddle team (has there been uptake? What barriers does the client face in accessing the service/ program);
- Checking in with the client to see how they are progressing toward a health or life goal;
- Completing an additional clinical screening tool;
- Referring to [Name] transportation and friendly visiting program where available;
- Accompanying the client to a community program or service; and
- Completing a [Name] safety assessment (e.g. home safety hazards, medication safety, food safety, falls).
