# Supplementary material for: Community Volunteers and Primary Care Providers Supporting Older Adults in System Navigation: A Mixed Methods Study
Source: Int J Integr Care. 2022 Mar 2;22(1):18. doi: 10.5334/ijic.5978 (PMC8896251; doi:10.5334/ijic.5978)
Supplement: Appendix C. — A sample Follow-up Report for a fictional client. [file ijic-22-1-5978-s3.pdf]

Appendix C: A sample Follow-up Report for a fictional client.

Follow-up [Program Name] Report: Janina Martell (1944-02-18)

Patient name: Janina Martell	Address:
Family Doctor: Dr. Singh	Date of last visit: 2020-01-22
**Reason for Follow-up Visit**	
☑ Facilitate connection to a specific community program	
**Volunteer Visit Summary**	
I was requested to visit Janina a second time to follow-up with her and share information about specific community fitness programs at the YMCA to support her goal in getting more exercise to improve her fitness level. I shared with her some different pamphlets that were publicly accessible on the YMCA website. We also opened up the YMCA website together, and she seemed very interested in Balance program (12-week exercise program for fall prevention). I provided Janina with the YMCA contact information, pamphlets, and she was very appreciative.	
**Volunteer Notes to Clinic**	
She did not register during our session, so I am not sure if she will attend the session. Overall, the visit went very well and Janina seemed excited about the program.	
**Health Care Team Summary of Review and Plan of Action**<br><br>**(*Optional: Huddle enters additional actions if applicable*)**	
Health care team to follow-up with client in 6 weeks about registration into programs.	
